# Alpha-Mangostin Ameliorates Bleomycin-Induced Pulmonary Fibrosis in Mice Partly Through Activating Adenosine 5′-Monophosphate-Activated Protein Kinase

**DOI:** 10.3389/fphar.2019.01305

**Published:** 2019-11-13

**Authors:** Ren-shi Li, Gong-hao Xu, Juan Cao, Bei Liu, Hai-feng Xie, Yuji Ishii, Chao-feng Zhang

**Affiliations:** ^1^State Key Laboratory of Natural Medicines, School of Traditional Chinese Medicines, China Pharmaceutical University, Nanjing, China; ^2^Sino-Jan Joint Lab of Natural Health Products Research, School of Traditional Chinese Medicines, China Pharmaceutical University, Nanjing, China; ^3^Research and Development Department, Chengdu Biopurify Phytochemicals Ltd., Chengdu, China; ^4^Laboratory of Molecular Life Sciences, Graduate School of Pharmaceutical Sciences, Kyushu University, Fukuoka, Japan

**Keywords:** alpha-mangostin, pulmonary fibrosis, myofibroblast differentiation, AMPK activation, NOX4

## Abstract

**Background:** Pulmonary fibrosis (PF) is a devastating interstitial lung disease and characterized by an abnormal accumulation of extracellular matrix (ECM). Nintedanib (NDN) and pirfenidone are two approved therapies for PF, but their potential side-effects have been reported. Recently, the use of natural supplements for PF is attracting attention. Alpha-mangostin (α-MG) is an active xanthone-type compound isolated from the nutritious fruit mangosteen.

**Purpose:** In the present study, the potential effect and underlying mechanism of α-MG were evaluated in bleomycin (BLM)-induced PF and activated primary lung fibroblasts (PLFs).

**Methods:** Histopathological changes and collagen deposition were analyzed *via* hematoxylin-eosin staining and Masson staining, the expression of nicotinamide adenine dinucleotide phosphate oxidase-4 (NOX4) involved in oxidative stress in lung tissues was analyzed by immunochemistry staining. The expressions of α-smooth muscle actin (α-SMA), collagen I (Col I), p-adenosine 5′-monophosphate-activated protein kinase (AMPK)/AMPK, and NOX4 were detected by Western blot, immunofluorescence or RT-PCR, and effects of α-MG on cell viability were detected using the 3-(4,5-dimethylthiazol-2-yl)-2,5-diphenyl tetrazolium bromide.

**Results:** *In vivo* results demonstrated that α-MG treatment (10 mg/kg/day) significantly ameliorated BLM-induced deposition of ECM in lung tissues. Moreover, α-MG could inhibit protein expressions of α-SMA and Col I as well as its mRNA levels. In addition, α-MG also significantly inhibited transforming growth factor-β1/Smad2/3 pathway and regulated the protein expression of matrix metalloproteinase-9 and tissue inhibitor of metalloproteinase-1 in lung tissues. *In vitro* results demonstrated that α-MG significantly increased p-AMPK/AMPK but reduced the protein expression level of α-SMA and Col I as well as NOX4 in activated PLFs. Further study demonstrated that these improvement effects were significantly blocked by compound C.

**Conclusion:** α-MG treatment significantly decreased oxidative stress in lungs partly by activating AMPK mediated signaling pathway in BLM-induced PF and activated PLFs and decreased the deposition of ECM. The present study provides pharmacological evidence to support therapeutic application of α-MG in the treatment of PF.

## Introduction

Pulmonary fibrosis (PF) is a chronic, progressive, and currently untreatable complication of several interstitial pulmonary diseases (Todd et al., 2012; Rafii et al., 2013). Clinical research demonstrated that prednisone, azathioprine, and N-acetylcysteine increased mortality in PF patients compared with the placebo (Raghu et al., 2012). Nintedanib (NDN) and pirfenidone are two approved therapies for PF, but their potential side-effects have been reported (Raghu et al., 2015). Recently, the use of natural supplements for PF is attracting attention (Lili et al., 2015; He et al., 2015; Xia et al., 2016).

Alpha-mangostin (α-MG) is a major xanthone-type compound in the peels of mangosteen (Nguemfo et al., 2009), which exhibits anti-inflammatory, anti-tumor (Hsieh et al., 2013; Franceschelli et al., 2016), anti-steatosis (Choi et al., 2015), and other biological activities (Chao et al., 2011; Chae et al., 2016). Although it has been reported that it can attenuate liver fibrosis (Khunvirojpanich et al., 2015), the therapeutic effect of α-MG on PF is not sure.

The fibrogenic process is a complex three-step pathophysiological process, including inflammatory responses, effector cell activation and migration, and excessive extracellular matrix (ECM) deposition. PF presents as excessive accumulation of ECM. Activated alveolar epithelial cells and myofibroblasts are the primary source of matrix components, which plays a critical role in the development of PF (Raghu, 2011). Elevated transforming growth factor-β1 (TGF-β1) levels are major inducers of PF (Leask and Abraham, 2004), direct blockade of TGF-β1 may be a strategy to suppress matrix protein synthesis, but this approach would produce many undesired systemic side effects because of the multiple biological activities of TGF-β1 (Pardali et al., 2010; Deelman and Sharma, 2015). Therefore, further characterization of the downstream signaling pathway of TGF-β1 in the generation of matrix proteins may provide an alternative anti-fibrotic strategy. Recent evidence suggests NAPDH oxidases-4 (NOX4) and AMP-activated protein kinase (AMPK) as potential therapeutic targets in PF (Amara et al., 2010; Li et al., 2015). NOX4 is expressed in pulmonary fibroblasts and targeting of NOX4 inhibited the fibrotic process in experimental PF (Chan et al., 2009; Hecker et al., 2009). AMPK is a metabolic regulator, the activation of it exhibits improvement in metabolic disorders and prevention of organ dysfunction during pulmonary diseases (Myerburg et al., 2010; Muanprasat et al., 2013; Zhao et al., 2014; Malik et al., 2015). What’s more, several studies have demonstrated that activation of AMPK effectively inhibited the over-expression of NOX4 in activated lung fibroblasts (Park et al., 2012; Jian et al., 2013; Li et al., 2015; Lu et al., 2015). However, whether the treatment of α-MG can target AMPK and NOX4 remains unknown. And after treatment of α-MG, the role of AMPK and related signaling pathway in the progression of PF remains to be further studied. Bleomycin (BLM) is a chemotherapeutic cancer agent, and PF is a primary side effect of clinic treatment. In present study, we evaluated the potential protective effect and underlying mechanism of α-MG in both BLM-induced PF and activated lung fibroblasts, which involved AMPK mediated signaling pathway. We demonstrated a beneficial function of α-MG in PF, and activating of AMPK by α-MG may be a therapeutic approach for the PF treatment.

## Materials and Methods

### Chemicals and Reagents

α-MG (purity over 99%) was prepared by Chengdu Biopurify Phytochemical Ltd. (Chengdu, China). BLM was obtained from Nippon Kayaku (Tokyo, Japan). NDN (40 mg/kg) was obtained from Shanghai Yuke Chemical Co., Ltd. (Shanghai, China). Recombinant TGF-β1 was purchased from PeproTech Inc. (Rocky Hill, NJ, USA). Compound C (CC) was provided by Selleck (Shanghai, China). 3-(4,5-Dimethylthiazol-2-yl)-2,5-diphenyl tetrazolium bromide (MTT) was obtained from Biosharp Technology Inc. (Hefei, China).

A Hydroxyproline (HYP) Assay Kit was purchased from Nanjing Jiancheng Bio-Engineering Institute (Nanjing, China). Antibodies against AMPK, phospho-AMPK (p-AMPK), TGF-β1, Smad2/3, and phospho-Smad2/3 (p-Smad2/3) were purchased from Cell Signal Technology Inc. (Massachusetts, USA). Antibodies against β-actin, alpha-smooth muscle actin (α-SMA), and horseradish peroxidase (HRP)-conjugated secondary antibody were purchased from Bioworld Technology Inc. (Dublin, OH, USA). An antibody against NOX4 was purchased from Abcam Technology Inc. (Cambridge, UK). An antibody against collagen-1 (Col I) was purchased from Wanleibio Technology Inc.

### Animals

Male C57/BL6 mice (6–8 weeks old, weighing 18–20 g) were supplied from the Comparative Medicine Centre of Yangzhou University (Yangzhou, China). Animals were cared for according to the General Recommendation and Provisions of the Chinese Experimental Animals Administration Legislation. The Institutional Ethical Committee of China Pharmaceutical University approved the animal protocol (SCXK, su, 2012–0004). Mice were housed in a climate-controlled room at 22 ± 2°C and 50 ± 10% humidity (12 h light/dark cycle), with free access to food and drinking water.

### Bleomycin-Induced Pulmonary Fibrosis in Mice

BLM-induced PF was induced as described previously (You et al., 2015). Briefly, mice were acclimated for 1 week and divided randomly into four groups. Mice were anaesthetized *via* intraperitoneal injection of a chloral hydrate solution (4%, 10 ml/kg) followed by intratracheal instillation of BLM (5 mg/kg) or saline as a control. One week after BLM administration, α-MG (10 mg/kg) was intragastrically administered daily for 14 consecutive days. NDN (40 mg/kg) was used as a positive control drug. The control groups and BLM groups received the same volume of 0.9% sterilized saline. Mice were euthanized on day 21 *via* an excessive intraperitoneal injection of chloral hydrate. Lung tissue was excised for lung coefficient measurement (lung weight/body weight; mg/g). The left lower lobes were fixed in 10% formalin for histopathological examination, and the remaining lung tissue was stored at −80°C.

### Histological Analysis

Lung tissues were fixed with 10% formalin, embedded in paraffin, sectioned, and stained with hematoxylin-eosin (H&E) or Masson’s trichrome. Experienced pathologists performed pathological evaluations in a single-blind manner. The specific scoring rules are as follows (Szapiel et al., 1979): 1) whether the alveolar wall is thickened, congested, and there is no hemorrhage or edema in the alveolar cavity; 2) presence or absence of fibrous tissue hyperplasia, emphysema, macrophage hyperplasia, or other cell proliferation; 3) bronchial epithelial cells with or without degeneration, necrosis and other diseases. According to the degree of light to heavy lesions: basic normal score is 0, slight lesions are recorded as 0.5, mild lesions are scored as 1 point, moderate lesions are scored as 2 points, and severe lesions are scored as 3 points. Sections were viewed using an Olympus BX53 microscope at 200× magnification.

### Measurement of Hydroxyproline Content

Approximately 40 mg of lung tissue homogenate was hydrolyzed in 1 ml of hydrolysate at 95°C for 20 min. HYP content in lung tissue was determined using commercial test kits according to the manufacturers’ instructions. HYP content was expressed as µg/mg.

### Immunohistochemistry and Immunofluorescence Staining

Paraffin-embedded lung sections (5 µm) were incubated with 3% H_2_O_2_ to eliminate endogenous peroxidase. Antigen retrieval was performed *via* heating, and non-specific binding sites were blocked using 5% skim milk in phosphate buffer saline (PBS) for 1 h. Sections were incubated with primary antibodies and second antibodies conjugated with HRP. Color was visualized *via* incubation of the sections with diaminobenzidine (DAB). Sections were viewed at 200× magnification.

Before immunofluorescence staining, cells were pretreated with or without the AMPK inhibitor CC (50 nM) for 1.5 h and subsequently in the absence or presence of TGF-β1 (10 ng/ml), α-MG (50 nM), or control [dimethylsulfoxide (DMSO)] for 48 h. Then, cells were fixed with 4% paraformaldehyde/PBS for 15 min, followed by incubation with 0.3% Triton X-100/PBS for 10 min, 2% bovine serum albumin (BSA)/PBS block solution for 2 h, and probed with primary antibody α-SMA (Bioworld Technology, Dublin, OH, USA; 1:200) overnight at 4°C. Cy3-conjugated secondary antibodies were used to amplify the signal. 4’,6-Diamidino-2-phenylindole (DAPI) was used to stain nuclei. Images were obtained by fluorescent microscopy (Olympus IX53).

### Cell Culture

Mouse primary lung fibroblasts (PLFs) were isolated as previously described (Tao et al., 2017). Briefly, PLFs outgrown from lung fragments were cultured in fibroblast growth media [Dulbecco’s modification of eagle’s medium (DMEM) with 10% fetal bovine serum (FBS) and penicillin-streptomycin] at 37°C in a 5% CO_2_ atmosphere. The percentage of fibroblasts was identified as 95% using morphology under microscope. Cells were detached with 0.25% trypsinization and seeded in 96-well plates at 4 × 10^4^ cells per well or six-well plates at 2 × 10^5^ cells per well. Cells were pretreated with or without the AMPK inhibitor CC (50 nM) for 1.5 h and subsequently in the absence or presence of TGF-β1 (10 ng/ml), α-MG (1–50 nM) or control (DMSO) for 48 h.

### Cell Viability Assay

Cell viability was assessed using the MTT assay. Briefly, cells were seeded in 96-well plates at 4×10^4^ cells per well and incubated in DMEM containing 10% FBS for 24 h. After pretreatment with or without drugs for 48 h, MTT (5 mg/ml) was added to the wells and incubated for an additional 4 h. The optical density was measured at 490 nm.

### Western Blot Analysis

Total proteins extracted from lung homogenates or cell lysates were lysed in ice-cold RIPA lysis buffer containing a 1:100 dilution of phenylmethanesulfonyl fluoride (PMSF, Beyotime, China). Protein concentrations were determined using the bicinchoninic acid (BCA) Protein Assay Kit (Beyotime, China). Samples were boiled for 10 min, equal amounts of protein (50 µg/lane) were separated using SDS-PAGE and transferred to polyvinylidene fluoride membranes (Merck Millipore, Billerica, USA). Membranes were probed with primary antibodies overnight at 4°C and incubated with HRP-conjugated secondary antibodies at 25°C for 2 h. Bands were visualized using high-sig electrochemiluminescence (ECL) detection reagent (Tanon, China).

### Real-Time Quantitative Polymerase Chain Reaction Analysis

Total RNA was extracted from lung tissues using TRIzol reagent (Invitrogen Life Technologies, USA), reverse-transcribed to complementary DNA (cDNA) using the TransScript First-Strand cDNA Synthesis Kit (Toyobo, Japan), and stored at −80°C. Relative gene expression was quantified using Q-PCR using SYBR^®^ Premix Ex Taq^TM^ (Takara, China) in StepOne^TM^ Real-Time PCR (Life Technologies, USA). Total RNA (0.5 µg) was reverse transcribed before the following PCR conditions were performed: 94°C for 2 min followed by 40 cycles at 94°C for 15 s, 58°C for 30 s, 72°C for 30 s, and final extension at 72°C for 10 min. **Table 1** shows the primer sequences (5′ to 3′), and data were quantified using the comparative Ct (ΔCt) method. The results are presented as the mean ratio to β-actin.

**Table 1:** Primers used for real-time polymerase chain reaction.

Gene		Product size (bp)	Product size (bp)
β-Actin	Forward	5′-CTGAGAGGGAAATCGTGCGT-3′	208
	reverse	5′-CCACAGGATTCCATACCCAAGA-3′	
NOX4	Forward	5′-CATTTGGAAGCCCATTTGAG-3′	119
	reverse	5′-TGGTTTCCAGTCATCCAGTAGAG-3′	
a-SMA	Forward	5′-CCACGAAACCACCTATAACAGC-3′	236
	reverse	5′-CCACGAAACCACCTATAACAGC-3′	
Col I	Forward	5′-CTGACTGGAAGAGCGGAGAG-3′	116
	reverse	5′-CGGCTGAGTAGGGAACACAC-3′	

### Statistical Analysis

All data were presented as mean ± SD. Comparisons between two groups were analyzed using Student’s *t*-test, and one-way ANOVA with Dunnett’s multiple comparison test was used for comparisons of multiple groups. Significance of survival curve was analyzed using the Kaplan-Meier method and log-rank test. Statistical analyses were performed using GraphPad Prism (San Diego, CA). *P*-values< 0.05 were considered to be statistically significant.

## Results

### Alpha-Mangostin Ameliorates Bleomycin-Induced Pulmonary Fibrosis in Mice

**Figure 1:**
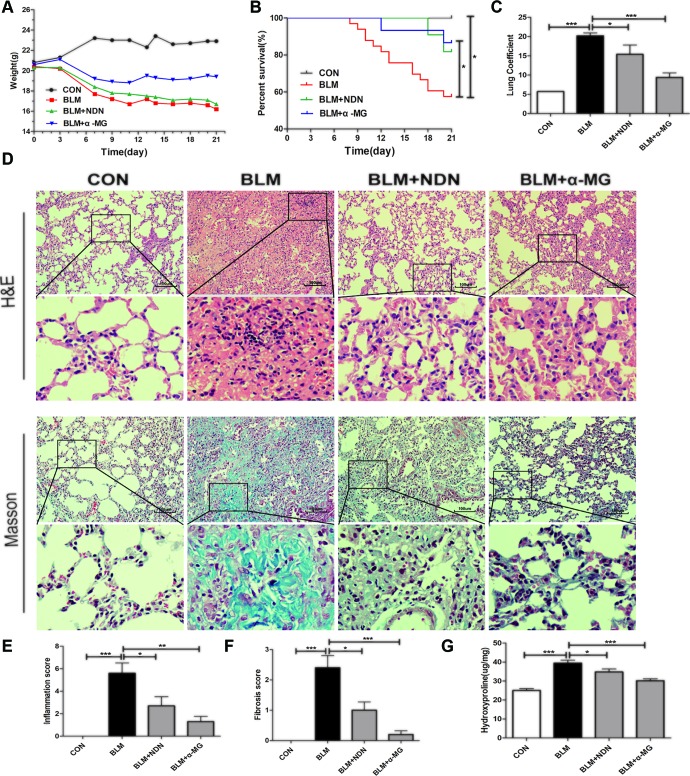
Alpha-mangostin (α-MG) treatment diminished bleomycin (BLM)-induced PF damage in mice. One week after BLM treatment (5 mg/kg), mice were intragastrically administered with α-MG (10 mg/kg) or nintedanib (40 mg/kg) once a day for 14 days. Mouse lungs were collected on day 21 after BLM treatment. The body weight **(A)**, survival rate **(B)**, pulmonary coefficient **(C)**, and hydroxyproline content **(G)** in lungs were determined, and the representative images (HE or Masson’s trichrome staining) of pulmonary histopathological changes in mice **(D)** and comparisons of the inflammation score **(E)** and fibrosis score **(F)** between the experimental groups are shown. Representative H&E and Masson’s trichrome-stained tissue sections of lungs under 200 × magnification. Data were expressed as the means ± SD (n = 9). *p < 0.05, **p < 0.01, ***p < 0.001, NS, non-significant.

BLM-induced murine PF is a useful experimental model of interstitial PF. C57/BL6 mice are highly susceptible to BLM-induced fibrosis (Lattaa et al., 2015). Therefore, C57/BL6 mice were selected to prove α-MG’s effectiveness on BLM-induced PF model. Day 7 after BLM administration is considered the beginning of the fibrotic phase with concomitant resolution of the acute inflammatory reaction. α-MG (10 mg/kg) was intragastrically administered at day 7 for 14 days after BLM (5 mg/kg) administration. BLM (16.2 g in average in day 21) significantly decreased mouse body weight compared with CON (22.9 g in average in day 21), but treatment with α-MG (10 mg/kg/day) (19.4 g in average in day 21) or NDN (positive drug, 40 mg/kg/day) (16.7 g in average in day 21) tended to not only prevent the body weight loss and survival rate reduction (**Figures 1A, B**), but also exhibited a significant decrease in the lung coefficient and HYP levels (**Figures 1C, G**). Qualitative examination of lung sections using H&E and Masson trichrome staining suggested that α-MG markedly ameliorated pulmonary histopathological changes and reduced the progression of fibrosis (**Figures 1D–F**).

### α-MG Reduces the Expression of Alpha-Smooth Muscle Actin and Collagen I in Vivo

**Figure 2:**
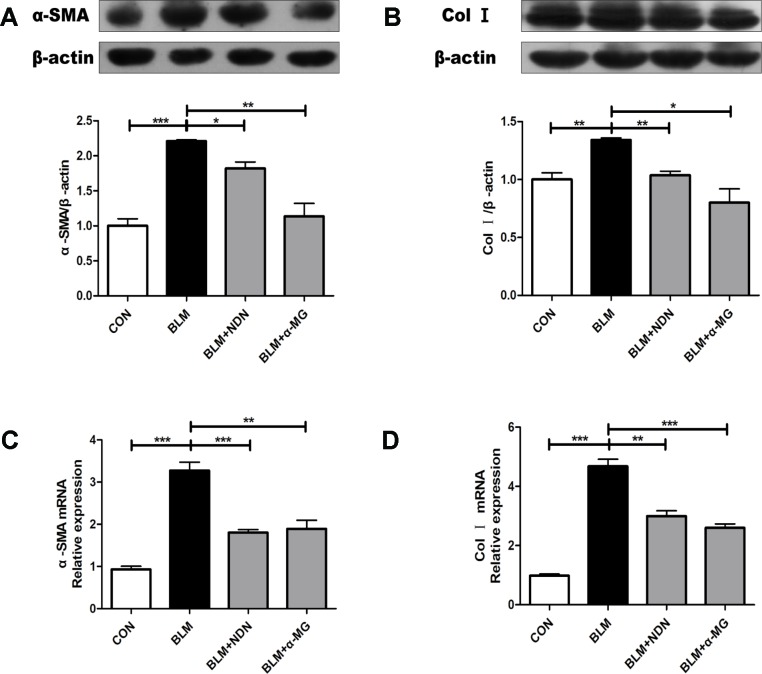
Alpha-mangostin (α-MG) reduced the expression of α-smooth muscle actin (α-SMA) and collagen I (Col I) in bleomycin (BLM)-induced PF. One week after BLM treatment (5 mg/kg), mice were intragastrically administered with α-MG (10 mg/kg) or nintedanib (40 mg/kg) once a day for 14 days. Mouse lungs were collected on day 21 after BLM treatment. Protein expression of α-SMA **(A)** and Col I **(B)** were detected using Western blot analysis. Relative mRNA expression of α-SMA **(C)**, Col I **(D)** were detected using real time-PCR analysis. Data were expressed as mean ± SD (n ≥ 3). *p < 0.05, **p < 0.01, ***p < 0.001, NS, non-significant.

The massive secretion of collagen by myofibroblasts is the main source of ECM, while α-SMA is a marker protein of myofibroblasts. As shown in **Figures 2A–D**, α-MG treatment dramatically reduced the expression of α-SMA and Col I both at mRNA and protein levels.

### α-MG Alleviates Metalloproteinase and Transforming Growth Factor-β1/smad2/3 Signaling Pathway

**Figure 3:**
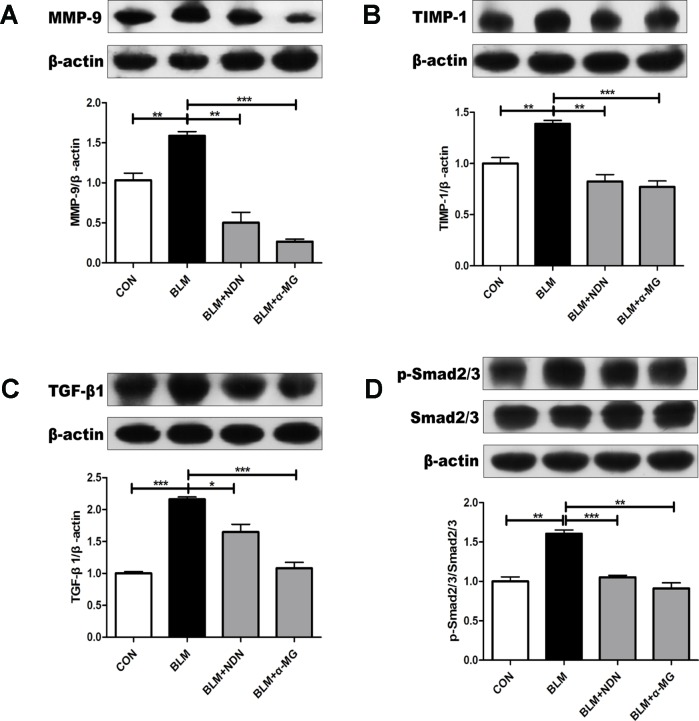
Alpha-mangostin (α-MG) blocked transforming growth factor-β1 (TGF-β1)/Smad2/3 signaling pathway and regulate MMPs/tissue inhibitor of metalloproteinase (TIMP) system balance in bleomycin (BLM)-induced pulmonary fibrosis. One week after BLM treatment (5 mg/kg), mice were intragastrically administered with α-MG (10 mg/kg) or nintedanib (40 mg/kg) once a day for 14 days. Mouse lungs were collected on day 21 after BLM treatment. Protein expression of MMP-9 **(A)**, TIMP-1 **(B)**, TGF-β1 **(C)**, and p-Smad2/3 and Smad2/3 **(D)** were detected using Western blot analysis. Data were expressed as mean ± SD (n ≥ 3). *p < 0.05, **p < 0.01, ***p < 0.001, NS, non-significant.

Increased deposition of ECM and reduced matrix degradation are the major characters of PF. Matrix metalloproteinase (MMPs) accelerate the degradation of ECM in PF lungs while tissue inhibitor of metalloproteinase (TIMPs) are endogenous inhibitor of MMPs and the balance of MMPs and TIMPs is critical to the steady state of ECM (Pardo et al., 2016). After the last drug administration, MMP-9 and TIMP-1 protein expressions were examined. We found an increase in both MMP-9 and TIMP-1 expression in BLM-damaged lung tissues, which were significantly alleviated by treatment with α-MG (**Figures 3A, B**). Additionally, TGF-β1/Smad2/3 is the most studied pro-fibrotic canonical pathway in the process of PF (Akhurst and Hata, 2012). Compared with the model group, α-MG treatments markedly reduced abnormal expression of TGF-βl expression and Smad 2/3 phosphorylation in lungs (**Figures 3C, D**).

### Alpha-Mangostin Promotes Adenosine 5′-Monophosphate-Activated Protein Kinase Activation and Ameliorates Oxidative Stress

**Figure 4:**
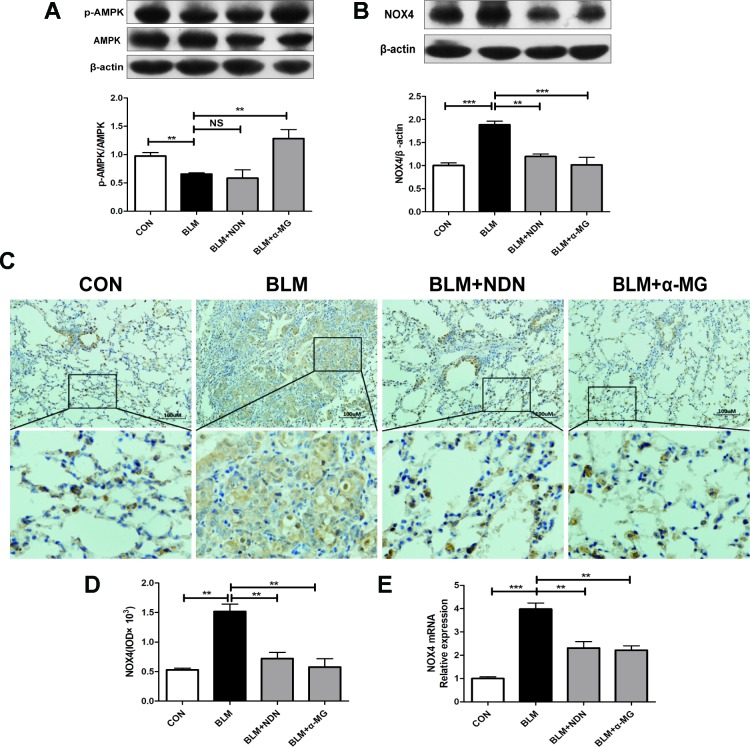
Alpha-mangostin (α-MG) promoted adenosine 5′-monophosphate-activated protein kinase (AMPK) activation and ameliorated oxidative stress *in vivo*. One week after bleomycin (BLM) treatment (5 mg/kg), mice were intragastrically administered with α-MG (10 mg/kg) or nintedanib (40 mg/kg) once a day for 14 days. Mouse lungs were collected on day 21 after BLM treatment. Expression of p-AMPK, AMPK **(A)**, and nicotinamide adenine dinucleotide phosphate oxidase-4 (NOX4) **(B)** in lung tissues were detected using Western blot analysis. Representative images showing NOX4 expression using immunochemistry under 200 × magnification **(C** and **D)**. NOX4 mRNA level was detected using real time-PCR analysis **(E)**. Data were expressed as the means ± SD (n ≥ 3). *p < 0.05, **p < 0.01, ***p < 0.001, NS, non-significant.

AMPK is a pivotal energy sensor that alleviates the process of fibrogenesis. Current studies identified that AMPK activation blocked increased NOX4 expression in a PF model (Park et al., 2012; Sato et al., 2016). In the present study, we also confirmed that α-MG treatments could significantly promote AMPK activation in lungs compared with the BLM group (**Figure 4A**)

Previous study has demonstrated a pivotal role for NOX4 in TGF-β1 signaling and myofibroblast differentiation (Sato et al., 2016). NOX4 is expressed in thickened pulmonary arteries in idiopathic PF (Pache et al., 2011; Cheresh et al., 2013; Ashish and Thannickal, 2016). As shown in **Figure 4B–E**, α-MG treatment markedly inhibited BLM-induced high expression and transcriptional level of NOX4 protein in lungs using Western blot analysis, immunochemistry staining, and real-time quantitative PCR analysis, suggesting α-MG can effectively ameliorate BLM-induced oxidative stress in lungs. Therefore, further research between AMPK activation and NOX4 inhibition following α-MG treatment is needed to elucidate this mechanism.

### Alpha-Mangostin Inhibited Trans-Differentiation of Lung Fibroblast Into Myofibroblasts *In Vitro*

**Figure 5:**
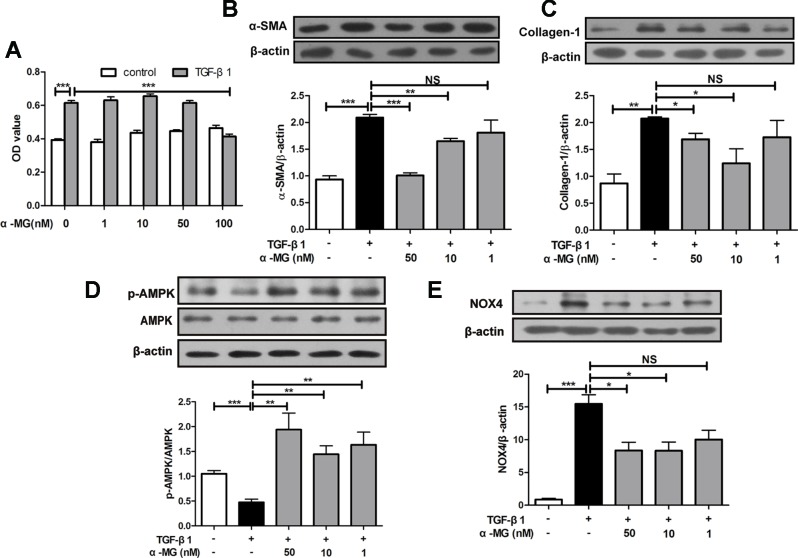
Alpha-mangostin (α-MG) inhibited transforming growth factor-β1 (TGF-β1)-induced lung fibroblasts differentiation *in vitro*. Mouse primary lung fibroblasts (PLFs) were treated with TGF-β1 (10 ng/ml) in the absence or presence of α-MG for 48 h. Inhibitory effects of α-MG (1–100 nM) on cell viability were detected using the MTT assay **(A)**. Protein expressions of α-smooth muscle actin **(B)**, collagen I **(C)**, p-adenosine 5′-monophosphate-activated protein kinase (AMPK), AMPK **(D)**, and nicotinamide adenine dinucleotide phosphate oxidase-4 **(E)** in PLFs after α-MG treatment (1–50 nM) were detected using Western blot analysis. Data were expressed as mean ± SD (n ≥ 3). *p < 0.05, **p < 0.01, ***p < 0.001, NS, non-significant.

BLM-induced PF model in mice is characterized by activated myofibroblasts (Bhattacharyya et al., 2013). Trans-differentiation of lung fibroblasts into myofibroblasts facilitated lung wound healing *via* synthesis of ECM during the repair process (Hinz et al., 2007; Wynn, 2011), and myofibroblasts are characterized by α-SMA expression and collagen synthesis (Klingberg et al., 2013). In the present study, mouse PLFs were treated with TGF-β1 (10 ng/ml), and fibrotic-related protein expression was determined through Western blot analysis. Cell viability of PLFs was investigated using the MTT assay. As shown in **Figure 5A**, α-MG could significantly inhibit the proliferation of activated PLFs at 100 nM, but no effect on the viability of normal or activated cells was observed at 1–50 nM. Then, α-MG (10 or 50 nM) significantly inhibited α-SMA and Col I expression (**Figures 5B, C**), suggesting that α-MG can significantly inhibit TGF-β1-induced trans-differentiation of lung fibroblast. In addition, α-MG also significantly activated AMPK but suppressed NOX4 overexpression in activated PLFs (**Figures 5D, E**)

### Alpha-Mangostin Inhibits Collagen Synthesis and Oxidative Stress by Activating Adenosine 5′-Monophosphate-Activated Protein Kinase

**Figure 6:**
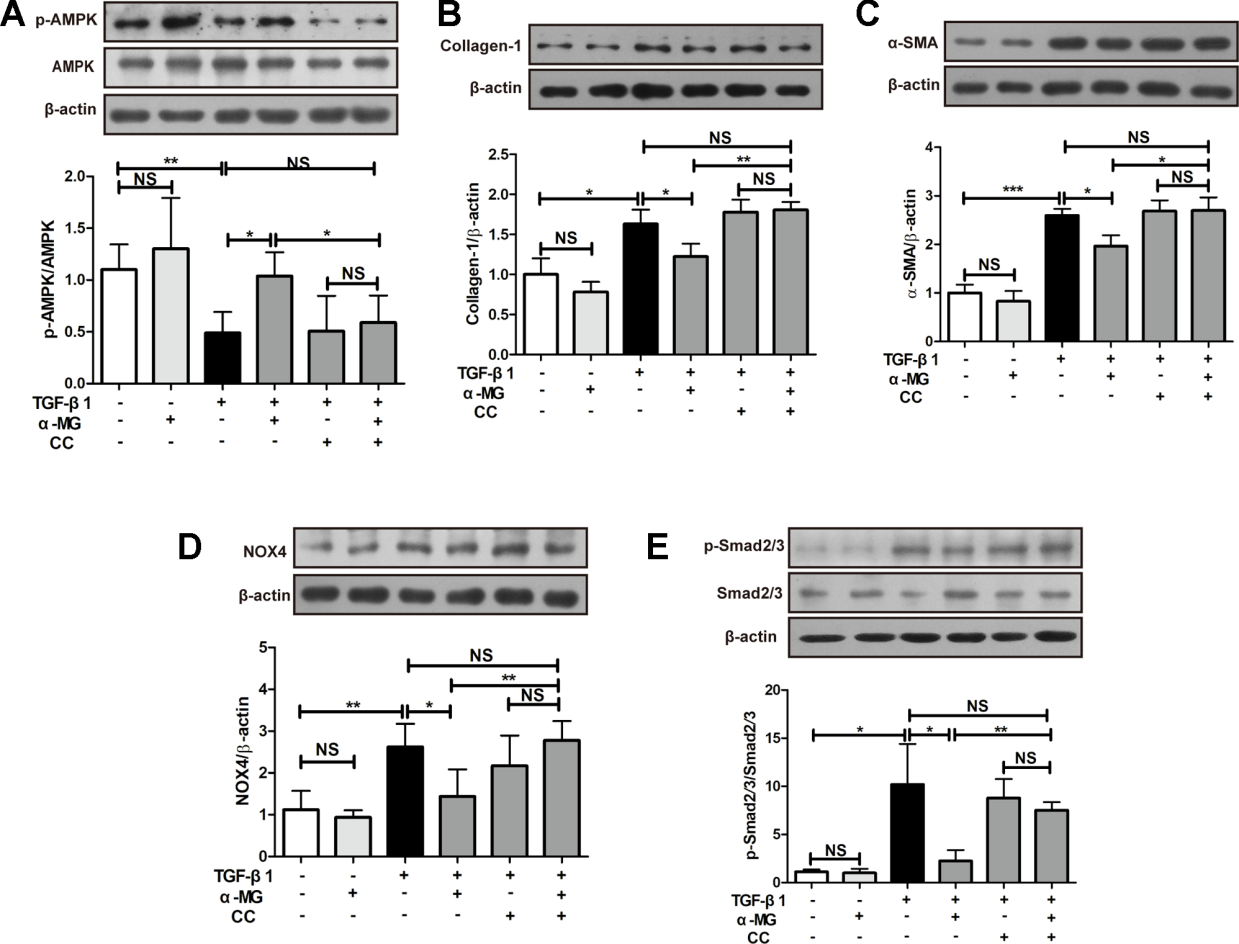
Alpha-mangostin (α-MG) exerted protective effects against lung myofibroblast differentiation *via* the targeting of adenosine 5′-monophosphate-activated protein kinase (AMPK) activation. Mouse primary lung fibroblasts were pre-treated with/without compound C (50 nM) for 1.5 h in the absence or presence of α-MG (50 nM) and transforming growth factor-β1 for 48 h. Expression of p-AMPK, AMPK **(A)**, collagen I **(B)**, α-smooth muscle actin **(C)**, nicotinamide adenine dinucleotide phosphate oxidase-4 **(D)**, p-Smad2/3 and Smad2/3 **(E)** were detected using Western blotting analysis. Data were expressed as mean ± SD (n ≥ 3). *p < 0.05, **p < 0.01, ***p < 0.001, NS, non-significant.

**Figure 7:**
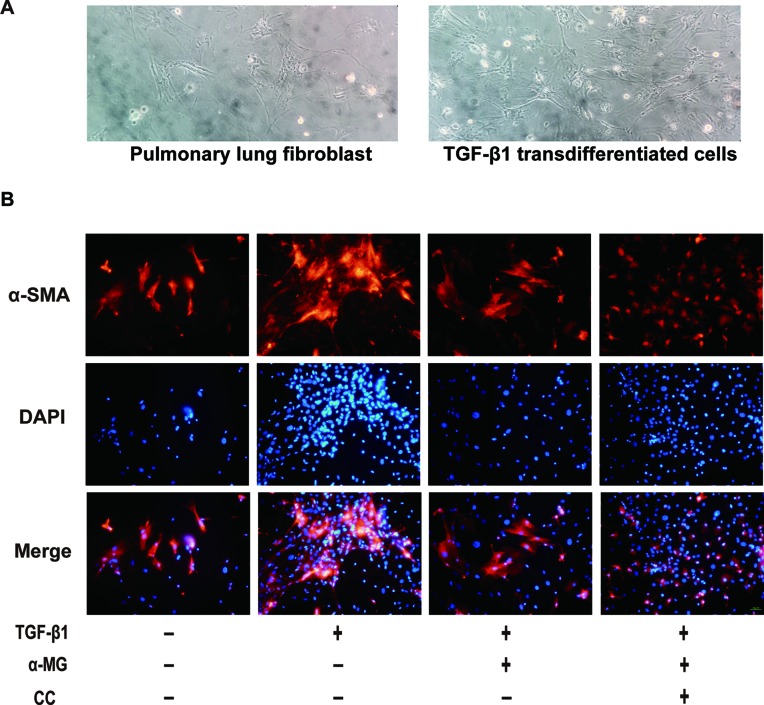
The morphology image of cells and immunofluorescence image of α-smooth muscle actin. The morphology of primary lung fibroblast and the transforming growth factor-β1 (TGF-β1) transdifferentiated cells were presented **(A)**. Immunofluorescence of α-smooth muscle actin (α-SMA) in TGF-β1 stimulated primary lung fibroblasts treated with alpha-mangostin (50 nM) and/or compound C (under 200 × magnification). Red, α-SMA, blue, 4’,6-diamidino-2-phenylindole **(B)**.

As shown in **Figure 6A**, α-MG (50 nM) could significantly promote AMPK activation in TGF-β1-activated PLFs which were significantly reversed by the co-treatment with an AMPK inhibitor (Compound C, CC). CC is an effective, reversible, selective AMPK inhibitor. It inhibits acetyl-CoA carboxylase (ACC) or metformin-induced ACC inactivation (Zhou et al., 2001), and inhibition of AMPK activity almost completely inhibits proteolysis of autophagy in HT29 cells (Meley et al., 2006). The activation of AMPK effectively alleviated inflammation-related fibrosis in lungs (Sunad et al., 2018). The suppression of α-MG on elevated protein expression of α-SMA and Col I were significantly blocked by CC in activated PLFs (**Figures 6B, C** and **Figure 7B**. Similarly, CC also significantly blocked the protection of α-MG on TGF-β1-stimulated NOX4 expression and Smad 2/3 phosphorylation (**Figures 6D, E**). Collectively, α-MG effectively inhibited TGF-β1-induced lung myofibroblast differentiation partly through activating AMPK.

## Discussion

α-MG is a xanthone-type active ingredient originally isolated from mangosteen with anti-inflammatory, anti-fungal, anti-tumor, anti-oxidant, cardio-protective, and anti-bacterial properties (Peres et al., 2000; Jindarat, 2014; Zhang et al., 2017). Recently study reported that α-MG showed potential anti-fibrotic effects in experimental liver fibrosis (Khunvirojpanich et al., 2015). In the present study, the effect of α-MG against BLM-induced PF in mice and the related molecular mechanism were investigated.

A previous study reported that α-MG exacerbates experimental colitis and promotes dysbiosis in mice (Gutierrez-Orozco et al., 2014), but another study showed α-MG ameliorates dextran sulfate sodium (DSS)-induced colitis through inhibition of nuclear factor kappa-B (NF-κB) and mitogen-activated protein kinase (MAPK) pathways (You et al., 2017). We speculated that these two opposite conclusions may be due to the different amounts of α-MG. So we examined the effect of α-MG on the colon of mice with BLM-induced PF, the result suggested that the treatment of α-MG at the dose of 10 mg/kg didn’t cause colonic lesions (**Supplementary Figure 1**). Besides, α-MG had no toxicity on normal mice at the dose of 10 mg/kg (**Supplementary Figure 2**). In conclusion, 10 mg/kg of α-MG for mice is a safe and effective dose.

Severe and progressive scar formation are important characteristics of PF (Barkauskas and Noble, 2014). Excessive lung scarring ultimately severely impairs gas exchange (Berend, 2014). In the BLM-induced PF model, the BLM alone group exhibited significantly elevated HYP levels and obvious collagen deposition in lungs when compared with the control group (**Figures 1** and **2**), but 1 week after BLM administration, α-MG treatments for 2 weeks significantly decreased lung inflammatory and fibrotic lesions in C57/BL6 mice (**Figure 1**). HYP is a key amino acid of collagen synthesis in fibrotic lung tissues (Yang et al., 2017). α-MG treatment not only significantly ameliorated the elevated HYP contents in lungs (**Figure 1**), but also inhibited BLM-induced high levels of TGF-β1, α-SMA and Col I as well as Smad2/3 phosphorylation in lungs (**Figures 2** and **3**). An imbalance in MMPs and TIMPs may result in fibrosis progression, and MMP-9 activates TGF-β1 and stimulates fibroblast contraction of collagen gels. α-MG treatments obviously reduced BLM-induced abnormal protein expression of MMP-9 and TIMP-1 in lung tissues (**Figure 3**), suggesting that α-MG could inhibit excessive accumulation of ECM during the process of BLM-induced PF in mice.

Several studies have confirmed there is a close relationship between AMPK and fibrogenesis, AMPK activation may be a possible treatment for PF (Myerburg et al., 2010; Lu et al., 2015; Jiang et al., 2017). The AMPK agonist metformin can down-regulate TGF-β1-induced NOX4 expression and attenuate BLM-induced experimental PF (Sato et al., 2016). Oxidative stress contributes to the development of PF, NOX4 activation prolongs Smad 2/3 phosphorylation in cardiac fibroblasts (Cucoranu et al., 2005). The targeting NOX4 with small interfering RNA (siRNA) also suppresses mouse PF (Hecker et al., 2009) and NOX4 expression is upregulated in lung fibroblasts from idiopathic PF patients (Amara et al., 2007). *In vitro* studies have demonstrated that xanthones exhibit anti-oxidant activity (Williams et al., 1995; Jung et al., 2006). Mangosteen extract can attenuate the metabolic disorders of high-fat-fed mice *via* AMPK activation (Chae et al., 2016), and α-MG induces autophagy of glioblastoma cell *via* AMPK activation (Chao et al., 2011). However, the precise role of AMPK activation by α -MG treatment in its anti-PF effects is not clearly tested. In the present study (**Figures 2**– **4**), we have confirmed that α -MG treatment can dramatically activate AMPK and inhibited NOX4 expression as well as TGF-β1/Smad2/3 signaling pathway in lung tissues compared with the BLM alone group. 

The aetiology of PF is not known, but the activation of fibroblasts is a seminal step in the process of PF with the subsequent release of pro-fibrotic factors and ECM proteins, activated fibroblasts are responsible for matrix protein production, which is modulated by TGF-β1 (Wynn, 2007). Lung fibroblast is believed to play an important role in lung tissues repair and regenerative process during the process of BLM-induced PF in mice. In the present study, the mouse PLFs were used to investigate the underlying anti-fibrotic mechanism of α-MG. α-MG treatment (50 nM) significantly inhibited the TGF-β1-induced abnormal expression levels of α-SMA, Col I, and NOX4 (**Figure 5**). Myofibroblast differentiation is a key element in PF diseases. AMPK inhibition by CC abolished effects of α-MG on TGF-β1-mediated collagen synthesis (α-SMA and Col I) and NOX4 expression (**Figures 6** and **7**), suggesting that α-MG effectively ameliorated trans-differentiation of lung fibroblasts into myofibroblasts through activating AMPK.

## Conclusions

Collectively, α-MG treatment exerted anti-fibrotic effects *via* the targeting of AMPK signaling, which reduced collagen accumulation, modulated the redox status in lung fibroblasts, and ameliorated BLM-induced PF in mice.

## Data Availability Statement

All datasets generated for this study are included in the article/**Supplementary Material**.

## Ethics Statement

The animal study was reviewed and approved by The Institutional Ethical Committee of China Pharmaceutical University.

## Author Contributions

In this paper, R-SL and G-HX designed the study and carried out experiments. JC and BL contributed to pharmacology experiments. YI provided the experimental background guidance. H-FX contributed to compound preparation. C-FZ as the corresponding author undertook the design of this project and logic train of thought.

## Conflicts of Interest

Author H-FX was employed by Chengdu Biopurity Phytochemicals Ltd.

The remaining authors declare that the research was conducted in the absence of any commercial or financial relationships that could be construed as a potential conflict of interest.

## Abbreviations

α-MG, alpha-mangostin; AMPK, adenosine 5′-monophosphate (AMP)-activated protein kinase; α-SMA, α-smooth muscle actin; ACC, acetyl-CoA carboxylase; BCA, bicinchoninic acid; BLM, bleomycin; BSA, bovine serum albumin; CC, compound C; Col I, collagen I; DAB, diaminobenzidine; DAPI, 4’,6-diamidino-2-phenylindole; DMEM, Dulbecco’s modification of Eagle’s medium; ECL, electrochemiluminescence; ECM, extracellular matrix; FBS, fetal bovine serum; H&E, hematoxylin-eosin; HRP, horseradish peroxidase; HYP, hydroxyproline; MAPK, mitogen-activated protein kinase; MMP-9, matrix metalloproteinase-9; MTT, 3-(4,5-dimethylthiazol-2-yl)-2,5-diphenyl tetrazolium bromide; NDN, nintedanib; NF-κB, nuclear factor kappa-B; NOX4, NAPDH oxidases-4; PBS, phosphate buffer saline; PF, pulmonary fibrosis; PLFs, primary lung fibroblasts; siRNA, small interfering RNA; TGF-β1, transforming growth factor-β1; TIMP-1, tissue inhibitor of metalloproteinase-1.
