# Supplementary material for: Alpha-Mangostin Ameliorates Bleomycin-Induced Pulmonary Fibrosis in Mice Partly Through Activating Adenosine 5′-Monophosphate-Activated Protein Kinase
Source: Front Pharmacol. 2019 Nov 13;10:1305. doi: 10.3389/fphar.2019.01305 (PMC6863977; doi:10.3389/fphar.2019.01305)
Supplement: Supplementary file 1 [file Image_1.pdf]

Supplementary Material

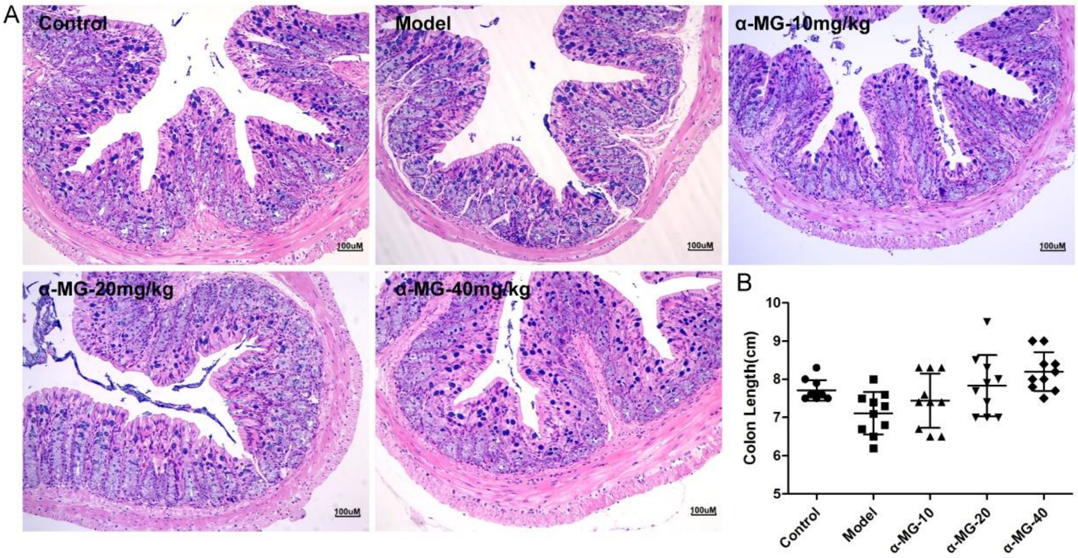

Fig. S1 Evaluate the effects of α-MG at the dose of 10 mg/kg on colonic lesions. One week after BLM treatment (5 mg/kg), mice were orally administered with α-MG (10, 20, 40 mg/kg) once a day for 14 days. Mouse lungs were collected on day 21 after BLM treatment. The representative H&E-stained colon sections (magnification × 100) (A) and colon length (B) from each group of mice were shown. Data were expressed as the mean ± S.D. (n = 10). NS, non-significant.
