# Supplementary material for: Alpha-Mangostin Ameliorates Bleomycin-Induced Pulmonary Fibrosis in Mice Partly Through Activating Adenosine 5′-Monophosphate-Activated Protein Kinase
Source: Front Pharmacol. 2019 Nov 13;10:1305. doi: 10.3389/fphar.2019.01305 (PMC6863977; doi:10.3389/fphar.2019.01305)
Supplement: Supplementary file 2 [file Image_2.pdf]

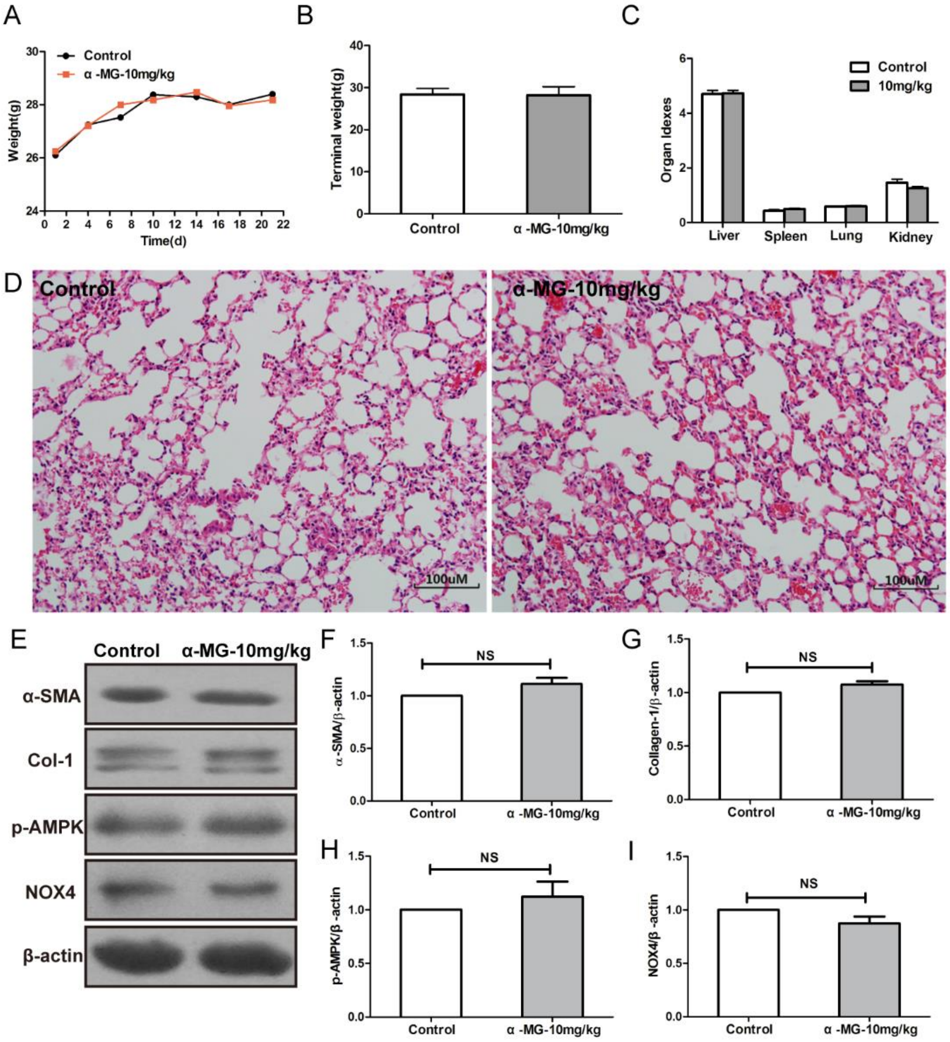

Fig. S2 Evaluate the toxicity of α-MG treatment on mice at the dose of 10 mg/kg. Mice were given an intragastric administration of α-MG (10 mg/kg) every day and they were euthanized on day 21. The body weight and terminal weight (A and B), organ index (C) were determined, and the representative images (H&E staining) among the experimental groups (D) were shown. Representative H&E-stained tissue sections of lungs under 200 × magnification. Protein expressions of α-SMA, Col I, p-AMPK, NOX4 (E-I) were detected by western blot analysis. Data were expressed as the mean ± S.D. (n = 9). NS, non-significant.
